# The spiritual distress assessment tool: an instrument to assess spiritual distress in hospitalised elderly persons

**DOI:** 10.1186/1471-2318-10-88

**Published:** 2010-12-13

**Authors:** Stefanie M Monod, Etienne Rochat, Christophe J Büla, Guy Jobin, Estelle Martin, Brenda Spencer

**Affiliations:** 1 Service of Geriatric Medicine & Geriatric Rehabilitation, University of Lausanne Medical Center (CHUV), 1011 Lausanne, Switzerland; 2 Chaplaincy Service, University of Lausanne Medical Center (CHUV), 1011 Lausanne, Switzerland; 3 Faculty of Theology and Religious Sciences, University of Laval, QC G1V 0A6 Quebec, Canada; 4 Institute of Social and Preventive Medicine (IUMSP), University Hospital Center and University of Lausanne, Bugnon 17, 1005 Lausanne, Switzerland

## Abstract

**Background:**

Although spirituality is usually considered a positive resource for coping with illness, spiritual distress may have a negative influence on health outcomes. Tools are needed to identify spiritual distress in clinical practice and subsequently address identified needs. This study describes the first steps in the development of a clinically acceptable instrument to assess spiritual distress in hospitalized elderly patients.

**Methods:**

A three-step process was used to develop the Spiritual Distress Assessment Tool (SDAT): 1) Conceptualisation by a multidisciplinary group of a model (Spiritual Needs Model) to define the different dimensions characterizing a patient's spirituality and their corresponding needs; 2) Operationalisation of the Spiritual Needs Model within geriatric hospital care leading to a set of questions (SDAT) investigating needs related to each of the defined dimensions; 3) Qualitative assessment of the instrument's acceptability and face validity in hospital chaplains.

**Results:**

Four dimensions of spirituality (Meaning, Transcendence, Values, and Psychosocial Identity) and their corresponding needs were defined. A formalised assessment procedure to both identify and subsequently score unmet spiritual needs and spiritual distress was developed. Face validity and acceptability in clinical practice were confirmed by chaplains involved in the focus groups.

**Conclusions:**

The SDAT appears to be a clinically acceptable instrument to assess spiritual distress in elderly hospitalised persons. Studies are ongoing to investigate the psychometric properties of the instrument and to assess its potential to serve as a basis for integrating the spiritual dimension in the patient's plan of care.

## Background

The relationship between spirituality and medicine is a field of growing interest [1-3]. In palliative care, the spiritual dimension is considered as an important component of care along with physical, psychological, and social or existential support [4]. Spirituality is also considered an essential component of the multidimensional approach used in geriatric care of elderly patients who face illness, disability, and potentially life-threatening events [5].

Spirituality has been shown to influence, usually in a positive way, coping with illness, disability, or life-threatening events [6-10]. Many studies have documented significant associations between spirituality and better mental, physical, and functional health, especially in cancer, HIV, and hospice patients [11,12]. Some studies have, however, shown that negative manifestations of spirituality may be associated with poorer health outcomes. Religious struggle, defined as negative feelings towards God, feeling punished by God, or believing that « the devil is at work in the illness », has been associated with increased mortality in elderly patients [13]. Spiritual distress, that can be defined as "a state in which the individual is at risk of experiencing a disturbance in his/her system of belief or value that provides strength, hope, and meaning to life" [14], seems also associated with more severe depression and desire for hastened death in end-of-life patients [15,16]. Spiritual distress might have a potentially harmful effect on patients' prognosis and quality of life [17-20].

Despite evidence suggesting an association between spiritual distress and worse health outcome, very few intervention studies have been conducted to improve patients' spiritual health [21,22]. This may be explained by the lack of consensus on the definition of spirituality, and, as a consequence, of spiritual distress, within health care research [23-25]. Numerous instruments have been developed to assess spirituality. Most currently available describe behaviours, beliefs or attitudes towards spirituality [26-28]. Although some instruments measuring spiritual well-being or spiritual needs might equally reflect spiritual distress [29-31], none of these instruments has been designed for this specific purpose. Moreover, conceptual models on which to base spiritual assessment, spiritual distress recognition and spiritual intervention in hospital settings are essentially lacking, and are called for in order to improve patient care [25,32]. These conceptual models should also be congruent with other Bio-Psycho-Social processes of care in order to promote integrative models of care in hospital settings. These shortcomings need to be addressed as a prerequisite to conducting intervention studies.

The present paper describes work to address this issue and presents: a) an operational definition of spiritual distress; b) the successive steps in the development of an instrument to assess spiritual distress in hospitalized elderly patients; c) the subsequent assessment of this instrument's face validity and acceptability in clinical practice.

## Methods

### Basic concepts

There are different ways to assess spirituality; this research focuses on assessment of the patient's *spiritual state*. Spiritual state is here defined as the patient's feelings regarding his or her spirituality. Spiritual state is dynamic: it fluctuates according to a hypothesised spectrum of spiritual wellness, ranging from spiritual well-being to spiritual distress. A spiritual state might be worse because of external stressors such as illness or bereavement; it may also be improved by spiritual intervention. This concept of spiritual state appeared as the most appropriate way to assess spirituality within the hospital setting. The intention is that assessment of a patient's spiritual state should serve to determine the need for specific interventions.

Based on this definition of a spiritual state, an operational definition of *spiritual distress *was hypothesised. The hypothesis was made that spiritual distress arises from unmet spiritual needs and that the greater the degree to which a spiritual need remains unmet, the greater the disturbance in spiritual state and the greater the level of spiritual distress experienced by the patient.

### Development of the Spiritual Distress Assessment Tool (Figure 1)

**Figure 1:**
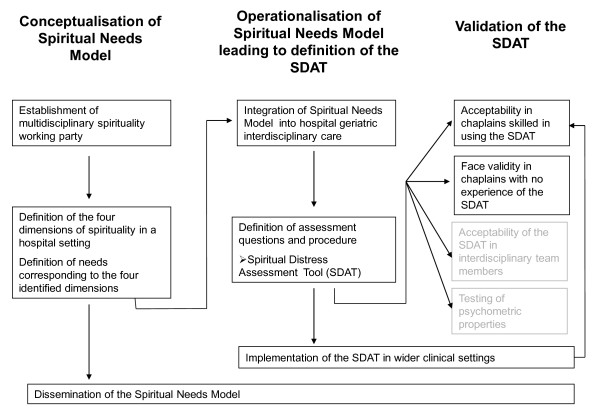
**Process of SDAT development and assessment of face validity and acceptability of the SDAT**.

The development of The Spiritual Distress Assessment Tool (SDAT) was based on a conceptual model of spiritual needs assessment previously published under the name of the Spiritual Needs Model [33].

Development of the Spiritual Distress Assessment Tool was yet carried out in three stages.

### a) Conceptualisation of spirituality and spiritual needs in hospitalised persons: definition of the Spiritual Needs Model [33]

An interdisciplinary group of health professionals (one physician, four nurses, and three chaplains), working in five different geriatric hospitals in Switzerland, met on fourteen occasions over a two-year period to define and conceptualise spirituality in the hospitalised person. The group was directed by one of the co-authors (ER).

A literature search and review in PubMed and Google, using "spirituality" and "religiosity" as search terms, was performed to select and define candidate dimensions that could characterize spirituality in hospitalised persons. Candidate dimensions were discussed and consensus was achieved through the sharing of spiritual care experiences, role play and case analysis. Finally, using the same process, the working party further defined the spiritual needs corresponding to each selected dimension of spirituality.

The work of the interdisciplinary group resulted in a definition of *spirituality in hospitalised persons*, of the *dimensions that characterize a patient' spirituality *and of the *needs corresponding to each of these dimensions*.

The overall concept was defined as **The Spiritual Needs Model **[33].

### b) Definition of the Spiritual Distress Assessment Tool (SDAT) and guidelines for administration

Two of the authors (SM and ER) decided to integrate the Spiritual Needs Model into hospital geriatric care over a six month period in order to assess its practicability in clinical care.

This phase of the research was conducted in the post-acute care unit of the Department of Geriatric Medicine, University of Lausanne Medical Center. This 66-bed unit admits patients aged 65 years and older and provides interdisciplinary care to restore the highest possible level of functional independence and quality of life. Eighty percent of patients report a Judaeo-Christian religious background.

During this phase, the leader of the working party (ER) was integrated into the interdisciplinary team. He performed systematic bedside assessments of patients' spirituality using the framework of the Spiritual Needs Model and participated in weekly interdisciplinary team meetings to share the results of this assessment with health professionals.

Over the six month period, 69 patients were assessed by the chaplain using the framework of the Spiritual Needs Model. Of those patients proposed a meeting with the chaplain, only one refused. Characteristics of the participants are described in Table 1.

**Table 1:** Patients' characteristics

Characteristics	Population (N = 69)
Age (years)	82.5 ± 8.3

Women (%)	78.3

Living alone (%)	62.5

Cognitive impairment* (%)	30.4

Depressive symptoms ^†^	12.1

Basic ADL at admission ^¥^	2.5 ± 1.6

* defined as a score < 24 at the Mini Mental State Examination (score ranging from 0 to 30, with higher scores indicating better cognition)

† defined as a score ≥ 6 at the 15-item Geriatric Depression Scale (score ranging from 0 to 15, with higher scores indicating more depressive symptoms)

**¥ **Katz's basic Activities of Daily Living (score ranging from 0 to 6, with higher score indicating better function)

Based on this experience, spiritual needs assessment with use of the Spiritual Needs Model was progressively structured and systematised. In the course of this process, a set of questions was gradually devised for use in the interview to investigate the patient's spiritual needs and guidelines to conduct spiritual needs assessment (e.g. patient's consent, confidentiality) were defined. In parallel, a structured analytical framework was developed to assess the severity of unmet spiritual needs, as manifested in the interview.

This process resulted in the definition of the SDAT, that is, a formalised assessment procedure to identify unmet spiritual needs, to score the degree to which spiritual needs remained unmet and to determine the presence of spiritual distress.

This part of the SDAT development was approved by the institutional Ethical Review Board of the University of Lausanne.

### c) Assessment of the face validity and acceptability of the SDAT

It was considered important to assess the validity of the SDAT. However, as no consensus exists regarding the definition of spirituality and the dimensions that characterize spirituality, no real "definitional standard" could be said to exist [34]. Thus, true assessment of the content validity of the SDAT against a gold standard would not have been possible. However, face validity, considered as being a particular type of content validity, was assessed. Face validity refers to whether persons not involved in the development of an instrument perceive it as measuring what it is deemed to measure [35]. In this case, we established whether hospital chaplains experienced in hospital pastoral care, who had not been in any way involved in the development or use of the SDAT, perceived the instrument as able to measure a patient's' spirituality.

#### Face validity of the SDAT in chaplains with no experience of the SDAT, but experienced in providing hospital pastoral care

In order to assess these characteristics, a moderated structured discussion group was conducted with chaplains practising at the chaplaincy of the University of Lausanne Medical Center (see Appendix) who were unfamiliar both with the Spiritual Needs Model and with the SDAT. Of the six chaplains invited, four protestant chaplains accepted the invitation, one declined and one catholic chaplain was not available. Participants were invited to watch a video of a pastoral interview using the SDAT and subsequently participated in a moderated discussion lasting two hours. Chaplains were asked to compare the interview with their own way of conducting a first pastoral encounter with a patient, to determine whether all dimensions of a patient's spirituality were addressed in the SDAT interview and to express their view on the structured approach used to assess a patient's spirituality in the hospital setting.

#### Acceptability of the SDAT in chaplains already skilled in using the SDAT

Assessment of *acceptability *is commonly made in health services research with a view to determining the potential impact of proposed services, since services can only be effective if delivered and taken-up as intended. It is therefore important during implementation to assess acceptability in both service providers and service users.

As the SDAT was specially designed to be used by chaplains, the acceptability of the SDAT was assessed in members of the chaplaincy of the University of Lausanne Medical Center who were already trained in use of the instrument. In this case, the aim was to ascertain to what extent these chaplains perceived the instrument as relevant to their work and to what extent they considered its use feasible within the hospital setting.

Assessment of acceptability of the SDAT's use in other interdisciplinary team members (medical and paramedical) has also been performed [36] and is to be published separately.

All four chaplains skilled in application of the SDAT, and working in different hospital departments, participated in two structured, moderated group discussions, each lasting two hours. Topics covered included: methods and level of appropriation of the SDAT by the chaplains; acceptability of the sets of questions proposed for the patient's interview; definition of skills necessary to identify and score unmet spiritual needs.

## Results

### a) Conceptualisation of spirituality and spiritual needs in hospitalised persons: definition of the Spiritual Needs Model (Table 2)

**Table 2:** Spiritual Needs Model: dimensions of spirituality and corresponding needs in hospitalized patients

Dimension of spirituality	Definition of dimension	Needs associated with dimension
**Meaning**	The dimension that provides orientation to an individual's life and promotes his or her overall life balance.	**The Need for life balance: **The need to rebuild a new life balance and the need to learn how to better cope with illness or disability.

**Transcendence**	An anchor point exterior to the person; the relationship with an external foundation that provides a sense of grounding. The group considered that everyone has an external foundation, even if different from God. For example, for some people, this transcendence might be found in nature, beauty, or art.	**The Need for connection**: The need for connection with his or her existential foundation and the need for Beauty (aesthetic sense).

**Values**	The system of values that determines goodness and trueness for the person; it is made apparent in the person's actions and life choices.	*The Need for values acknowledgement: The need that health professionals know and respect one's values. *The Need to maintain control: The need to understand and to feel included in decision-making processes and to be associated with health professionals' decisions and actions.

**Psycho-social Identity**	The patient's environment; those elements, such as society, caregivers, family, and close relationships that together make up the person's singular identity.	**The Need to maintain identity**: The need to be loved, to be heard, to be recognized, to be in touch, to have a positive image of oneself and to feel forgiven.

*According to the hospital setting, two different needs were clearly distinguished to translate the values dimension.

Overall, spirituality in the hospitalised elderly person was defined as the particular coherence expressed when describing one's meaning of life, referring to one's transcendence and explaining one's values.

Spirituality, in the particular context of hospital setting, was defined as a multidimensional concept that includes four dimensions considered to be interrelated: *Meaning*, *Transcendence*, *Values *and *Psycho-social Identity*.

The *Meaning *dimension was defined as that which provides orientation to an individual's life and promotes his or her overall life balance.

The *Transcendence *dimension was defined as an anchor point exterior to the person; the relationship with an external foundation that provides a sense of grounding.

The *Values *dimension was defined as the system of values that determines goodness and trueness for the person, as made apparent in his or her actions and life choices.

The *Psycho-social Identity *dimension was defined as the patient's environment; those elements, such as society, caregivers, family, and close relationships that together make up a person's singular identity.

In hospital care, the patient's medical, psychological and social needs are systematically defined, assessed and addressed. The same approach has therefore been applied regarding the patient's spiritual needs. Needs corresponding to each dimension of spirituality were thus defined.

The four defined dimensions of spirituality and their corresponding needs are summarized in Table 2.

### b) Definition of the SDAT (Additional file 1: Table S1)

Using the Spiritual Needs Model, a set of questions was developed to facilitate investigation of the patient's needs (Additional file 1: Table S1). These questions serve as prompts to be used only if the patient does not spontaneously mention anything related to the investigated need.

#### Guidelines for administering the SDAT

The SDAT is administered according to the following procedure:

First, in order to identify unmet spiritual needs, a 20-30 minute semi-structured interview is conducted by the chaplain with the patient after having obtained his/her *consent*.

Second, immediately following the interview, the chaplain conducts an assessment of how the patient spoke about his or her five spiritual needs, using the analytical framework to determine the eventual presence of spiritual distress.

Third, needs are scored on a 4-point Likert scale ranging from 0 (no unmet spiritual need) to 3 (severe unmet spiritual need). A global score of spiritual distress may therefore range from 0 (no spiritual distress) to 15 (severe spiritual distress). Examples of statements made by patients experiencing unmet needs are provided in Table 3.

**Table 3:** Examples of statements made by patients experiencing unmet needs

Need for life balance	"I know I've got to find a way to cope, but I just can't manage. I just don't have the strength any longer"
**Need for connection**	"I think that God has abandoned me"; "I am no longer able to paint and it was the painting that kept me in touch with the force that kept me going".

**Need for values acknowledgement**	"I'm just a number here. The staff doesn't know who I am."

**Need to maintain control**	"I don't know what I'm doing here in the hospital. Why hasn't anyone given me any medicine?"

**Need to maintain identity**	"My friends don't come and visit me; my family has no idea of what I'm going through here; I just don't know myself any longer."

At the end of the interview, the chaplain tells the patient precisely what information he or she intends to relay to the interdisciplinary team members, and requests the patient's consent to proceed. When presenting results of spiritual assessments to the team, special attention is taken to avoid unnecessarily sharing intimate information and to ensure *confidentiality*.

### c) Assessment of the face validity and acceptability of the SDAT

#### Face validity of the SDAT in chaplains with no experience of the SDAT, but experienced in hospital pastoral care

Chaplains reported overall positive appraisal of the SDAT.

The *Meaning*, *Transcendence *and *Psychosocial Identity *dimensions were clearly acknowledged by this group. The *Values *dimension and, in particular, its related needs (need to maintain control; need for values acknowledgement) were more debated. The group mostly acknowledged that chaplains do not systematically address this dimension unless they perceive some discomfort around these issues for the patient. Nevertheless, they generally agreed that this dimension was part of the patient's spirituality, as it reflects the patient's need to make meaningful life choices. Some chaplains also commented that certain aspects of religiosity, such as connection with the faith community and the need for ritual, should have been more clearly assessed in the video interview. They considered that these aspects should not simply be subsumed under the *Transcendence *dimension, but viewed as an additional dimension.

Their appraisal of the structured format for spiritual assessment differed from that of the group of chaplains skilled in using the SDAT (see below). They raised the question of the overall goal of a pastoral interview; for most, it is to engage with the patient and not to assess or to evaluate disturbance in their spiritual health. They argued that a spiritual interview should be tailor-made for each patient, and should, therefore, be less directive or restrictive than the SDAT. Some reluctance was expressed about using a structured format, as this seemed to imply that spirituality could be reduced to an assessment instrument. They perceived a risk of "medicalising" [37,38] spirituality and limiting its assessment to a health perspective. Nevertheless, it was agreed that the structured format of the SDAT would be of use when integrating pastoral care into health care and could help chaplains synthesise their evaluation and transmit meaningful information to health professionals. The group also agreed that a structured format could stimulate the assessment of dimensions that are not systematically addressed by chaplains, such as the *Values *dimension.

#### Acceptability of the SDAT in chaplains already skilled in using the SDAT

Overall, the SDAT was perceived by the chaplains as a useful adjunct to their pastoral interviews. They reported that the instrument allowed for more precise assessment of patients' spiritual needs. They also reported that the SDAT facilitated the communication of their observations to health professionals, and helped them clarify their potential role as well as their own implication in patient care. The structured format of the SDAT emerged as both an advantage (more efficient use of time, better organisation of the interview, systematic investigation of the four dimensions) and a disadvantage (restricts the flexibility of the interview, conveys the impression that spirituality can be "put in a box"). The group related that they tended to use the SDAT when asked by health professionals to visit a patient, the instrument allowing for a better synopsis and transfer of relevant information.

The set of standardised SDAT questions for the patient's interview was considered as acceptable and appropriate by the group. Chaplains felt comfortable enough with the instrument to consider potential useful applications in other settings or with younger patients (assuming that additional questions such as how they saw their future in terms of work, family life, or children were used).

Chaplains considered that it was not difficult to identify unmet spiritual needs during the patient interview. In contrast, assessing the *severity *of unmet spiritual needs proved more problematic. In particular, they pointed to the issue of adequately distinguishing between the severity of unmet spiritual needs and the availability of resources to cope with these needs. A patient with a severe unmet need for life balance may either be with or without resources to face this unmet need (e.g., he may or not have a good social network). The chaplains noted that when coping resources were absent, they tended to score more highly the level of spiritual distress than when these resources were present.

Numerous skills necessary to use the SDAT were outlined by the group, the most important being good communication skills, such as the ability to build a meaningful relationship with the patient before going ahead with the semi-structured interview, and having empathetic listening skills. A second group of required skills was more related to knowledge, such as familiarity with the four spiritual dimensions and their related spiritual needs, and theological and pastoral skills. A third group of skills included the capacity to analyse and synthesise the interview, and, a fourth group, the capacity to transmit relevant information to other team members.

## Discussion

This paper presents in detail the different steps in the development of an instrument aimed to assess spiritual distress in hospitalised older persons.

Overall, results show that the proposed conceptualisation of spirituality in hospitalised elderly patient as defined in the Spiritual Needs Model and the corresponding assessment instrument (SDAT) have face validity in chaplains providing hospital pastoral care and prove acceptable to those experienced in their application. Furthermore, chaplains did not report a feeling of confusion with psychological assessment, a criticism sometimes made of other spirituality constructs [39]. Certain reservations were, however, expressed.

Some chaplains felt that the definition of the different dimensions was somewhat unusual. The *Meaning *dimension is widely recognised as central components of spirituality [40,41]. In the literature, *Meaning *generally refers to the finding of a global meaning to life and death, and is generally associated with purpose in life [3]. Elderly patients often mention that because of their "old age", they have no purpose in life, but still see meaning in life. Thus, the definition of *Meaning *given in the Spiritual Needs Model refers to what provides orientation to an individual's life and promotes his or her overall life balance, rather than to definitions of the individual's new projects. Special attention was also given to the *Values *dimension. This dimension is less frequently identified as a specific dimension of spirituality. However, this dimension was warranted by the importance of recognising each patient's personal values so as to ensure respect for the patient's autonomy, dignity and integrity [42,43]. This was considered especially important in the hospital setting, given the vulnerability of elderly patient in this respect.

Second, some chaplains would have attributed a stronger, more explicit, place to religious practice, considering, for example, that rituals should be viewed as an additional spirituality dimension. Third, an additional important reservation concerned the *raison d'être *of pastoral hospital work: several chaplains expressed their concern that pastoral care could become medicalised and then be seen as a simple adjunct to health care. These reservations will need to be adequately addressed when attempting to further integrate spirituality assessment and management into routine care.

Interestingly, chaplains did not challenge our initial assumption that spiritual distress results from unmet spiritual needs. In fact, chaplains commonly recognized that the most promising way to integrate spirituality into health care is to be consistent with the other care processes established by the interdisciplinary team and the established institutional policy. This approach implies that the same approach be applied to spiritual needs as to bio-psycho-social needs. It seemed therefore rational to define spiritual distress as unmet needs.

Although not formally assessed, information obtained from patient contact during the development process indicates the feasibility and acceptability to patients of a systematic and structured bedside assessment of their spirituality. Also, the chaplain's participation in weekly interdisciplinary team meetings to share the results of his assessment has demonstrated the feasibility of integrating spirituality assessment into routine interdisciplinary geriatric care. A survey enquiring about interdisciplinary team members' appraisal of systematic spirituality assessment was conducted and showed that the contribution of the chaplain to improving patient care through weekly team meetings was considered essential [36].

It is, however, acknowledged that the feasibility and acceptability demonstrated is context-specific. Whether similar acceptance will be observed in other settings requires further study. This work was performed in a clinical setting already familiar with a comprehensive approach to patients' needs; these conditions may prove to be a pre-requisite for successful integration of spirituality assessment and for the participation of chaplains in routine care. The Christian origin and advanced age of patients enrolled in this phase of the development probably facilitated the acceptability of the encounter with the chaplain. Further assessment of acceptability will therefore be needed in larger, more diverse, elderly populations.

Besides these acknowledged limitations, the present work also has several strengths. The SDAT was developed according to a rigorous structured process: spirituality in hospitalized older patients was conceptualized through a consensus process, and its dimensions and their corresponding needs were then specified. The model was subsequently implemented within a clinical setting in order to operationalize further the assessment process. This process, going from the definition of spirituality to the definition of an instrument to assess spirituality, has previously been adopted in the development of other spirituality assessment instruments (e.g. The spirituality Index of Well-Being [44,45]) and strengthens the relevance of the instrument. Finally, face validity and acceptability in experienced chaplains were assessed. Though relatively long and complex, this approach had the advantage of ensuring contextual relevance for the instrument since issues regarding implementation could be dealt with progressively and *in situ*.

Although the SDAT was developed specifically in a population of hospitalized elderly patients, chaplains working with different populations saw considerable potential for use in other settings and in other age groups. Our procedure of assessment (a semi-structured interview) enables the patient to speak about spirituality with his or her own words and from very different perspectives. This should ensure relevancy of the SDAT for every patient, whatever their age or religious or spiritual background. Ultimately, the quality and limitations of the SDAT will be judged by the sustainability and dissemination of its use: by other chaplains, in other Departments and institutions, in research and evaluation, and, ultimately, in different cultural and religious contexts. Furthermore, as previously mentioned, the instrument's use is conditional on the availability of staff experienced in interdisciplinary care and with access to appropriate training facilities.

As yet, very few instruments have been developed on the basis of a spiritual needs construct. Two instruments, coming from nursing research, have been identified [31,46]. These two instruments were based on qualitative studies of patients who were asked to describe their specific spiritual needs. The approach presented here is unique because spiritual needs were assessed on the basis of a previously defined concept of spirituality. This structured approach ensures coherence between theoretical work and the investigative process.

## Conclusions

These preliminary results suggest that the SDAT is an acceptable instrument to assess spiritual distress in hospitalised persons. The instrument provides a tool for communication between disciplines, based on a shared vocabulary, and provides a new basis for integrating spirituality into the patient's plan of care. Further research is underway to assess the SDAT's acceptability in a larger sample of elderly patients and to investigate its psychometric properties. These are necessary steps before its application in intervention studies; that is, before using the SDAT to assess the impact of spiritual distress on health outcomes and patient prognosis.

## Appendix

### The chaplaincy of the University of Lausanne Medical Center

This chaplaincy was created by the hospital management together with both the Catholic and Protestant churches and has responsibility for pastoral care within the hospital and for hospital pastoral training.

Chaplains work in all departments of the hospital, regardless of the patient's religious affiliation.

The chaplaincy is composed of 7 ordained chaplains (2 Catholic; 5 Protestant) and 5 lay chaplains (4 Catholic; 1 Protestant).

External chaplains from other religious affiliations (rabbis, imams, Greek orthodox priests) are solicited on patient request.

## Competing interests

The authors declare that they have no competing interests.

## Authors' contributions

SM planned the study, supervised the development of the tool, supervised the validation procedure, and wrote the paper. ER conceptualized the tool, and helped write the paper. CB helped planned the study and contributed to revising the paper. GJ contributed to conceptualization of the tool and revising the paper. EM contributed to revising the paper. BS conceptualized the overall qualitative methodology, performed the validation and revised the manuscript.

All authors read and approved the final manuscript.

## Pre-publication history

The pre-publication history for this paper can be accessed here:

http://www.biomedcentral.com/1471-2318/10/88/prepub

## Supplementary Material

Additional file 1 ***Table S1: *Structure of the Spiritual Needs Model and the Spiritual Distress Assessment Tool**. Click here for file

## References

[B1] Thoresen CE Harris AHS Spirituality and health: What's the evidence and what's needed? Ann Behav Med 2002 24 3 13 10.1207/S15324796ABM2401_02 12008792

[B2] Miller WR Thoresen CE Spirituality, religion, and health: An emerging research field Am Psychol 2003 58 24 35 10.1037/0003-066X.58.1.24 12674816

[B3] Vachon M Fillion L Achille M A conceptual analysis of spirituality at the end of life J Palliat Med 2009 12 53 59 10.1089/jpm.2008.0189 19284263

[B4] Sulmasy DP A biopsychosocial-spiritual model for the care of patients at the end of life Gerontologist 2002 42 Special Issue 3 24 33 12415130 10.1093/geront/42.suppl_3.24

[B5] Monod S Rochat E Bula C Michael T Evans Walker, Emma D Walker Is there a place for spirituality in the care of elderly patients? Religion and psychology 2009 New York: Novapublishers

[B6] Koenig HG Pargament KI Nielsen J Religious coping and health status in medically ill hospitalized older adults J Nerv Ment Dis 1998 186 513 521 10.1097/00005053-199809000-00001 9741556

[B7] Kirby SE Coleman PG Daley D Spirituality and Well-Being in Frail and Nonfrail Older Adults J Gerontol B Psychol Sci Soc Sci 2004 3 P123 P129 10.1093/geronb/59.3.p123 15118015

[B8] Crowther MR Parker MW Achenbaum WA Larimore WL Koenig HG Rowe and Kahn's model of successful aging revisited: positive spirituality -- the forgotten factor Gerontologist 2002 42 613 620 12351796 10.1093/geront/42.5.613

[B9] Krause N Religious meaning and subjective well-being in late life J Gerontol B Psychol Sci Soc Sci 2003 3 S160 S170 10.1093/geronb/58.3.s160 12730317

[B10] Idler EL Kasl SV Religion among disabled and nondisabled persons II: attendance at religious services as a predictor of the course of disability J Gerontol Soc Sci 1997 52 6 S306 S316 10.1093/geronb/52b.6.s306 9403524

[B11] Koenig HG McCullough ME Larson DB Handbook of religion and health 2001 New York: Oxford University Press

[B12] Koenig HG Larson DB Larson SS Religion and coping with serious medical illness Ann Pharmacother 2001 35 352 359 10.1345/aph.10215 11261534

[B13] Pargament KI Koenig HG Tarakeshwar N Hahn J Religious struggle as a predictor of mortality among medically ill elderly patients: a 2-year longitudinal study Arch Intern Med 2001 161 15 1881 1885 10.1001/archinte.161.15.1881 11493130

[B14] Carpenito-Moyet Nursing diagnosis: application to clinical practice 2004 10 Philadelphia: Lippincott Williams & Wilkins

[B15] McClain CS Rosenfeld B Breitbart W Effect of spiritual well-being on end-of-life despair in terminally-ill cancer patients Lancet 2003 361 1603 1607 10.1016/S0140-6736(03)13310-7 12747880

[B16] Rodin G Lo C Mikulincer M Donner A Gagliese L Zimmermann C Pathways to distress: the multiple determinants of depression, hopelessness, and the desire for hastened death in metastatic cancer patients Soc Sci Med 2009 68 562 569 10.1016/j.socscimed.2008.10.037 19059687

[B17] Grant E Murray SA Kendall M Boyd K Tilley S Ryan D Spiritual issues and needs: perspectives from patients with advanced cancer and nonmalignant disease. A qualitative study Palliat Support Care 2004 2 371 378 10.1017/S1478951504040490 16594399

[B18] Monod S Rochat E Martin E Bula C Spiritual assessment in older patients undergoing post-acute rehabilitation: A pilot study Gerontologist 2007 47 S774

[B19] Astrow AB Wexler A Texeira K He MK Sulmasy DP Is failure to meet spiritual needs associated with cancer patients' perceptions of quality of care and their satisfaction with care? J Clin Oncol 2007 25 5753 5757 10.1200/JCO.2007.12.4362 18089871

[B20] Hills J Paice JA Cameron JR Shott S Spirituality and distress in palliative care consultation J Palliat Med 2005 8 782 788 10.1089/jpm.2005.8.782 16128652

[B21] Miller DK Chibnall JT Videen SD Duckro PN Supportive-Affective Group Experience for persons with life-threatening illness: reducing spiritual, psychological, and death-related distress in dying patients J Palliat Med 2005 8 333 343 10.1089/jpm.2005.8.333 15890044

[B22] Tarakeshwar N Pearce MJ Sikkema KJ Development and implementation of a spiritual coping group intervention for adults living with HIV/AIDS: A pilot study Mental Health, Religion & Culture 2005 8 179 190

[B23] Moberg DO Assessing and measuring spirituality: Confronting dilemmas of universal and particular evaluative criteria J Adult Dev 2002 9 47 60 10.1023/A:1013877201375

[B24] Sloan RP Bagiella E VandeCreek L Hover M Casalone C Jinpu HT Should physicians prescribe religious activities? N Engl J Med 2000 342 25 1913 1916 10.1056/NEJM200006223422513 10861331

[B25] Vivat B Measures of spiritual issues for palliative care patients: a literature review Palliat Med 2008 22 859 868 10.1177/0269216308095990 18755826

[B26] Sinclair S Pereira J Raffin S A thematic review of the spirituality literature within palliative care J Palliat Med 2006 9 464 479 10.1089/jpm.2006.9.464 16629575

[B27] Mularski RA Dy SM Shugarman LR Wilkinson AM Lynn J Shekelle PG A systematic review of measures of end-of-life care and its outcomes Health Serv Res 2007 42 1848 1870 10.1111/j.1475-6773.2007.00721.x 17850523 PMC2254566

[B28] Stefanek M McDonald PG Hess SA Religion, spirituality and cancer: current status and methodological challenges Psychooncology 2005 14 450 463 10.1002/pon.861 15376283

[B29] Brady MJ Peterman AH Fitchett G Mo M Cella D A case for including spirituality in quality of life measurement in oncology Psychooncology 1999 8 417 428 10.1002/(SICI)1099-1611(199909/10)8:5<417::AID-PON398>3.0.CO;2-4 10559801

[B30] Ellison CW Spiritual well-being: Conceptualization and measurement J PsycholTheol 1983 11 330 340

[B31] Taylor EJ Prevalence and associated factors of spiritual needs among patients with cancer and family caregivers Oncol Nurs Forum 2006 33 729 735 10.1188/06.ONF.729-735 16858453

[B32] Brennan M Heiser D Introduction: Spiritual Assessment and Intervention: Current Directions and Applications J Religion Spirituality Aging 2004 17 1 20 10.1300/J496v17n01_01

[B33] Monod S Rochat E Bula C Spencer B The Spiritual Needs Model: Spirituality Assessment in the Geriatric Hospital Setting J Religion Spirituality Aging 2010 22 271 282 10.1080/15528030.2010.509987

[B34] Stewart AL Wonca Classification Committee Psychometric Considerations in Functional Status Instruments Functional Status Measurement in Primary Care 1990 New York: Springer-Verlag 3 26

[B35] Anastasi A Psychological Testing 1968 Toronto, Canada: The Macmillan Company

[B36] Monod S Rochat E Martin E Bula C Spirituality in post-acute rehabilitation: Appraisal by interdisciplinary team members J Am Gerioatr Soc 2008 56 S4 S110

[B37] Illich I The medicalization of life J Med Ethics 1975 1 73 77 10.1136/jme.1.2.73 809583 PMC1154458

[B38] Conrad P The medicalization of society: On the transformation of human conditions into treatable disorders 2007 Baltimore, MD, US: Johns Hopkins University Press

[B39] Moreira-Almeida A Koenig HG Retaining the meaning of the words religiousness and spirituality: a commentary on the WHOQOL SRPB group's "a cross-cultural study of spirituality, religion, and personal beliefs as components of quality of life" Soc Sci Med 2006 63 843 845 10.1016/j.socscimed.2006.03.001 16650515

[B40] Koenig HG Larson DB Matthews DA Religion and psychotherapy with older adults J Geriatr Psychiatr 1996 29 155 184

[B41] Blazer D Spirituality and aging well Generations: J Am Soc Aging 1991 15 61 65

[B42] Kemp P Rendtorff JD Mattsson N Bioethics and biolaw Vols 1 and 2 Copenhague: Rhodos 2000

[B43] Muldoon M King N Spirituality, health care, and bioethics Journal of Religion & Health 1995 34 329 349 10.1007/BF02248742 11660133

[B44] Daaleman TP Kuckelman CA Frey BB Spirituality and well-being: an exploratory study of the patient perspective Soc Sci Med 2001 53 1503 1511 10.1016/S0277-9536(00)00439-1 11710425

[B45] Daaleman TP Frey BB Wallace D Studenski S The Spirituality Index of Well-Being: Development and testing of a new measure J Fam Pract 2002 51 11 952 12485549

[B46] Hermann C Development and testing of the spiritual needs inventory for patients near the end of life Oncol Nurs Forum 2006 33 737 744 10.1188/06.ONF.737-744 16858454

