# Supplementary material for: The spiritual distress assessment tool: an instrument to assess spiritual distress in hospitalised elderly persons
Source: BMC Geriatr. 2010 Dec 13;10:88. doi: 10.1186/1471-2318-10-88 (PMC3017043; doi:10.1186/1471-2318-10-88)
Supplement: Additional file 1 — Table S1: Structure of the Spiritual Needs Model and the Spiritual Distress Assessment Tool. [file 1471-2318-10-88-S1.DOC]

**Table S1:** Structure of the Spiritual Needs Model and the Spiritual Distress Assessment Tool

| **SPIRITUAL NEEDS MODEL** | | **SPIRITUAL DISTRESS ASSESSMENT TOOL (SDAT)** | | |
| --- | --- | --- | --- | --- |
|  |  | **PATIENT INTERVIEW** | **INTERVIEW ANALYSIS** | |
| **Spiritual dimension** | **Need associated with the spiritual dimension** | **Set of questions for patient interview** | **Questions for analysing the interview and identifying unmet spiritual need** | **Scoring of unmet spiritual need (range from 0 to 3*)** |
| **MEANING**  Overall life balance | **NEED FOR LIFE BALANCE**  - need to maintain and/or rebuild an overall life balance  - need to learn to “live with” an illness or disability | Does your hospitalisation have any repercussions on the way you live usually?  Is your overall life balance disturbed by what is happening to you now (hospitalisation, illness)?  Are you having difficulties coping with what is happening to you now (hospitalisation, illness)? | How does the patient speak about his or her need for life balance?  Is the overall life balance of this patient disturbed? | To what degree does the *Need for Life Balance* remain unmet?   - 0 - 1 - 2 - 3 |
| **TRANSCENDENCE**  Anchor point exterior to the person | NEED FOR CONNECTION  - need for Beauty  - need to be connected with the personal existential anchor | Do you have a religion, a particular faith or spirituality?  Does what is happening to you now change your relationship to God /or to your spirituality? (closer to God, more distant, no change)  Is your religion / spirituality / faith challenged by what is happening to you now?  Does what is happening to you now change or disturb the way you live or express your faith / spirituality / religion? | How does the patient speak about his or her need for connection?  Is his or her need for connection disturbed? | To what degree does the *Need for Connection* remain unmet?   - 0 - 1 - 2 - 3 |
| **VALUES**  System of values that determine goodness and trueness for the person; the system is made apparent in the person’s actions and life choices | NEED FOR VALUES ACKNOWLEDGEMENT  - need that caregivers understand what has value and significance in his or her life  **NEED TO MAINTAIN CONTROL**  - need to understand and be involved in caregivers’ decisions and actions | Do you think that the health professionals caring for you know you well enough?  Do you have enough information about your health problem, and on the goals of your hospitalisation and treatment?  Do you feel that you are participating in the decisions made about your care?  How would you describe your relationship with the doctors and other health professionals? | How does the patient speak of his or her need that caregivers understand what has value and significance in his or her life?  How does the patient speak of his or her need to understand and be involved in caregivers’ decisions and actions? | To what degree does the *Need for Values Acknowledgement* remain unmet?   - 0 - 1 - 2 - 3   To what degree does the *Need to Maintain Control* remain unmet?   - 0 - 1 - 2 - 3 |
| **PSYCHO-SOCIAL IDENTITY**  The environment (society, caregivers, family, close relations) that maintain the person’s particular identity. | **NEED TO MAINTAIN IDENTITY**  - need to be loved, to be recognised  - need to be listened to  - need to be in contact (in particular with the person’s faith community and other people)  - need to have a positive self-image  - need to feel forgiven, to be reconciled | Do you have any worries or difficulties regarding your family or other persons close to you?  How do people close to behave with you now? Does it correspond with what you expected from them?  Do you feel lonely?  Could you tell me about the image you have of yourself in your current situation (illness, hospitalisation)?  Do you have any links with your faith community? | How does the patient speak of his or her need to maintain identity? | To what degree does the *Need to Maintain Identity* remain unmet?   - 0 - 1 - 2 - 3 |

* **0** = no evidence of unmet spiritual need; **1**= some evidence of unmet spiritual need; **2**= substantial evidence of unmet spiritual need; **3** = evidence of severe unmet spiritual need
